# The combination of trehalose and glycerol: an effective and non-toxic recipe for cryopreservation of human adipose-derived stem cells

**DOI:** 10.1186/s13287-020-01969-0

**Published:** 2020-10-31

**Authors:** Tian-Yu Zhang, Poh-Ching Tan, Yun Xie, Xiao-Jie Zhang, Pei-Qi Zhang, Yi-Ming Gao, Shuang-Bai Zhou, Qing-Feng Li

**Affiliations:** 1 Department of Plastic & Reconstructive Surgery, Shanghai Ninth People’s Hospital, Shanghai Jiao Tong University School of Medicine, 639 Zhizhaoju Road, Shanghai, 200011 People’s Republic of China; 2 College of Life Sciences, Shanghai Normal University, Shanghai, People’s Republic of China

**Keywords:** Adipose-derived stem cells, Trehalose, Glycerol, Cryoprotective agent

## Abstract

**Background:**

Adipose-derived stem cells (ADSCs) promote tissue regeneration and repair. Cryoprotective agents (CPAs) protect cells from cryodamage during cryopreservation. Safe and efficient cryopreservation of ADSCs is critical for cell-based therapy in clinical applications. However, most CPAs are used at toxic concentrations, limiting their clinical application.

**Objective:**

The aim of this study is to develop a non-toxic xeno-free novel CPA aiming at achieving high-efficiency and low-risk ADSC cryopreservation.

**Methods:**

We explored different concentrations of trehalose (0.3 M, 0.6 M, 1.0 M, and 1.25 M) and glycerol (10%, 20%, and 30% v/v) for optimization and evaluated and compared the outcomes of ADSCs cryopreservation between a combination of trehalose and glycerol and the commonly used CPA DMSO (10%) + FBS (90%). All samples were slowly frozen and stored in liquid nitrogen for 30 days. The effectiveness was evaluated by the viability, proliferation, migration, and multi-potential differentiation of the ADSCs after thawing.

**Results:**

Compared with the groups treated with individual reagents, the 1.0 M trehalose (Tre) + 20% glycerol (Gly) group showed significantly higher efficiency in preserving ADSC activities after thawing, with better outcomes in both cell viability and proliferation capacity. Compared with the 10% DMSO + 90% FBS treatment, the ADSCs preserved in 1.0 M Tre + 20% Gly showed similar cell viability, surface markers, and multi-potential differentiation but a significantly higher migration capability. The results indicated that cell function preservation can be improved by 1.0 M Tre + 20% Gly.

**Conclusions:**

The 1.0 M Tre + 20% Gly treatment preserved ADSCs with a higher migration capability than 10% DMSO + 90% FBS and with viability higher than that with trehalose or glycerol alone but similar to that with 10% DMSO + 90% FBS and fresh cells. Moreover, the new CPA achieves stemness and multi-potential differentiation similar to those in fresh cells. Our results demonstrate that 1.0 M Tre + 20% Gly can more efficiently cryopreserve ADSCs and is a non-toxic CPA that may be suitable for clinical applications.

## Background

Adipose-derived stem cells (ADSCs) constitute a subset of mesenchymal stem cells (MSCs) obtained from adipose tissue that has self-renewal and potential plasticity characteristics [1]. Various studies have demonstrated that ADSCs are highly promising for therapeutic use in regenerative medicine due to their immunomodulation, anti-inflammatory, and angiogenesis properties [2]. However, the tissue acquisition procedure requires surgery, which causes pain and results in an economic burden on the patients. Moreover, the number and quality of ADSCs decrease with age. ADSC therapy could be more flexible and acceptable if cells could be harvested during a single surgical procedure at a young age and then stored for future use. The long-term preservation of ADSCs is essential to this process.

Cryopreservation is a common method used for the long-term preservation of cells and tissues. Studies have shown that the addition of cryoprotective agents (CPAs) avoids the damaging effect of intracellular crystallization during freezing and thawing. However, dimethyl sulfoxide (DMSO) combined with fetal bovine serum (FBS) has the risk of toxicity and zoonotic infection [3, 4]. A highly efficient, non-toxic, and xeno-free CPA is required for clinical application.

Trehalose, which is a non-permeable disaccharide, is non-toxic and biodegradable [5]. Moreover, trehalose has a high glass transition temperature and can form a unique protective film on the cell surface to prevent osmotic shock during freezing and thawing [6]. However, the impenetrability of the cell membrane to trehalose limits its cryopreservation effect [7, 8]. To overcome this obstacle and improve the efficiency of trehalose, several experimental approaches have been tested [9–11], but minimal progress has been achieved in terms of clinical applications.

Glycerol is a permeable CPA that can stabilize the cell membrane and improve the viscosity of water inside and outside the cell [12, 13]. We hypothesized that the combination of glycerol and trehalose can be more efficient in protecting cells from cryodamage and maintaining cell viability and, thus, may be a more efficient formula for the clinical cryopreservation of ADSCs. In our study, we first selected the most optimum concentration of trehalose and glycerol when applied alone and then evaluated the effect of a combination of trehalose and glycerol on preserving ADSCs’ viability and cell function.

## Materials and methods

### Adipose tissue acquisition

Abdominal subcutaneous adipose tissues were obtained from 10 healthy female donors within the age range of 40–45 years who underwent abdominal liposuction. The donors all provided informed consent. This study was approved by the Ethics Committee of Shanghai Ninth People’s Hospital and complied with the principles of the Declaration of Helsinki.

### ADSC isolation and culture

The ADSCs were obtained by collagenase digestion as previously described [14]. Suspensions of ADSCs were cultured in DMEM/F12 containing 10% FBS (Gibco, Life Technologies, Grand Island, NY, USA) and 1% penicillin/streptomycin (GE Healthcare Life Sciences, Freiburg, Germany) and incubated at 37 °C in an atmosphere containing 5% CO_2_ and 95% humidity; this passage was P0. Every 2–3 days, the complete medium was changed. Upon reaching 80–90% confluency, the cells were passaged at a ratio of 1:3 and subcultured for three passages (P3). During the passaging process, 3 ml 0.25% trypsin-EDTA (Sigma, St. Louis, MO, USA) was used to digest the adherent ADSCs until the cells were shed from the Petri dish, and then, 3 ml DMEM/F12 containing 10% FBS and 1% penicillin/streptomycin was added to terminate digestion. Invitrogen™ Countess™ II FL (Thermo Fisher, Waltham, USA) was used to assess the cell concentration.

### CPA preparation

For the CPA preparation, we used the following methods: (i) trehalose (Tre) group: trehalose powder (Solarbio, China) was diluted with phosphate buffer saline (PBS) for the powder reconstitution according to the required concentrations (0.3 M Tre, 0.6 M Tre, 1.0 M Tre, and 1.25 M Tre) and filtered using a 0.22-μm filter; (ii) glycerol (Gly) group: glycerol (Hercules, Bio-Rad Laboratories, CA, USA) was diluted with PBS to yield the required concentration (10% Gly, 20% Gly, and 30% Gly); (iii) trehalose and glycerin group: 1.0 M Tre + 20% Gly, as previously described, trehalose powder was diluted with PBS and then diluted with glycerol according to our needs; (iv) 10% DMSO (Sigma-Aldrich, Santa Clara, CA, USA) + 90% FBS as a positive control; and (v) control group: only PBS without trehalose and glyceron as a negative control. At least 30 ml of the CPA (30 vials) was preserved per group.

### Cell cryopreservation and thawing

Approximately 1 × 10^6^ ADSCs (P3) were resuspended in 1 ml CPAs and transferred into cryovials (Thermo Fisher, Waltham, MA, USA). The cryovials were frozen in a Nalgene® Mr. Frosty freezing container (Thermo Fisher, Waltham, MA, USA) at a cooling rate of 1 °C/min to − 80 °C, stored overnight, and transferred into liquid nitrogen for 30 days for storage. For thawing, the cryovials were placed in a water bath at 37 °C under gentle shaking until the ice was completely melted. The thawed ADSCs were rinsed with 10 ml PBS by centrifugation at 1500 rpm for 5 min (2 times) using a Microfuge 20/20R centrifuge (Beckman, USA) and resuspended in 5 ml Dulbecco’s modified Eagle’s medium (DMEM)/F12 containing 10% fetal bovine serum (FBS) and 1% penicillin/streptomycin for further assessment.

### Assessment of cell viability

Trypan blue (Thermo Fisher, Waltham) staining was used to evaluate the cell viability. One milliliter of resuspended ADSCs was mixed with an equal volume of trypan blue, and the viability was assessed using an Invitrogen™ Countess™ II FL (Thermo Fisher, Waltham, USA).

### Assessment of cell proliferation

A cell counting kit-8 assay (Beyotime Biotechnology Company, Shanghai, China) was used to assess cell proliferation. Briefly, the ADSCs were plated onto 96-well plates (5000 cells/well) and cultured for 24 h, 48 h, and 72 h. Then, 10 μl CCK-8 solution was added to the culture medium, and the cells were incubated at 37 °C for 4 h. The colorimetric assessment was performed at 450 nm using a Thermo Scientific microplate reader (Thermo Fisher, Waltham, MA, USA) to obtain an optical density (OD) value representing ADSC proliferation.

### Assessment of cell morphology

The morphology of the ADSCs (5 × 10^5^ cells/ml) in the different CPA groups was estimated before cryopreservation and 72 h after the thaw culturing under a microscope.

### Assessment of cell migration

Post-thaw ADSCs were plated in 6-well plates (5 × 10^5^ cells/well) and incubated at 37 °C until 90% confluency was reached. Then, the cells were incubated in a medium without serum, and a 200-μl pipette tip was used to create a scratch on the cell monolayer; debris was removed, and the edge of the scratch was smoothed using PBS. The cells were photographed immediately (0 h) and after 12 h and 24 h. We measured the area of the wound using ImageJ software, and this area was denoted A0. After 12 h and 24 h, we measured the residual area of the wound. The level of migration was assessed by the ratio of the closure area to the initial wound area as follows: migration area (%) = (A0 − An)/A0 × 100, where A0 represents the initial wound area, and An represents the residual area of the wound at the end point (*t* = *n h*).

### Assessment of cell markers

For the flow cytometric analysis, post-thaw P3 ADSCs and fresh P3 ADSCs were incubated with monoclonal antibodies against CD90 (PE, BD Biosciences, USA), CD73 (APC, BD Biosciences), CD105 (PE, BD Biosciences), CD34 (PE, BD Biosciences), CD45 (PE, BD Biosciences), CD14 (BV421, BD Biosciences), and HLA-DR (FITC, BD Biosciences). Unstained cells were used to exclude autofluorescence and control the intensity of the background. The cells were subsequently washed with PBS and analyzed using a FACS Aria-flow cytometer (Becton-Dickinson, San Jose, CA, USA).

### Assessment of multi-lineage differentiation

The multi-lineage differentiation capacity of the ADSCs was detected as described by the International Society for Cellular Therapy [15]. Briefly, the cells were cultured in adipogenic induction medium for the designated duration and stained with Oil Red-O staining. To evaluate osteogenic differentiation, the cells were cultured in osteogenic-inducing medium for the designated duration and stained with alizarin red S. Chondrogenic differentiation was performed using the micromass culture technique; the cells were maintained in chondrogenic medium for up to 5 weeks and stained with Alcian blue. The adipogenic, osteogenic, and chondrogenic differentiation kits were obtained from Cyagen.

### Statistical analysis

All data were collected from at least three independent replications. The numerical data are presented as the mean ± standard deviation (SD). The statistical analyses were performed using GraphPad Prism8 software (version 6.01 software). The group differences were analyzed using a two-tailed Student’s *t* test or one-way analysis of variance (ANOVA), and the differences were considered significant at *p* < 0.05.

## Results

### The CPA 1.0 M Tre + 20% Gly significantly improves the viability of post-thaw ADSCs compared to trehalose and glycerol alone, similar to 10% DMSO + 90% FBS

**Fig. 1:**
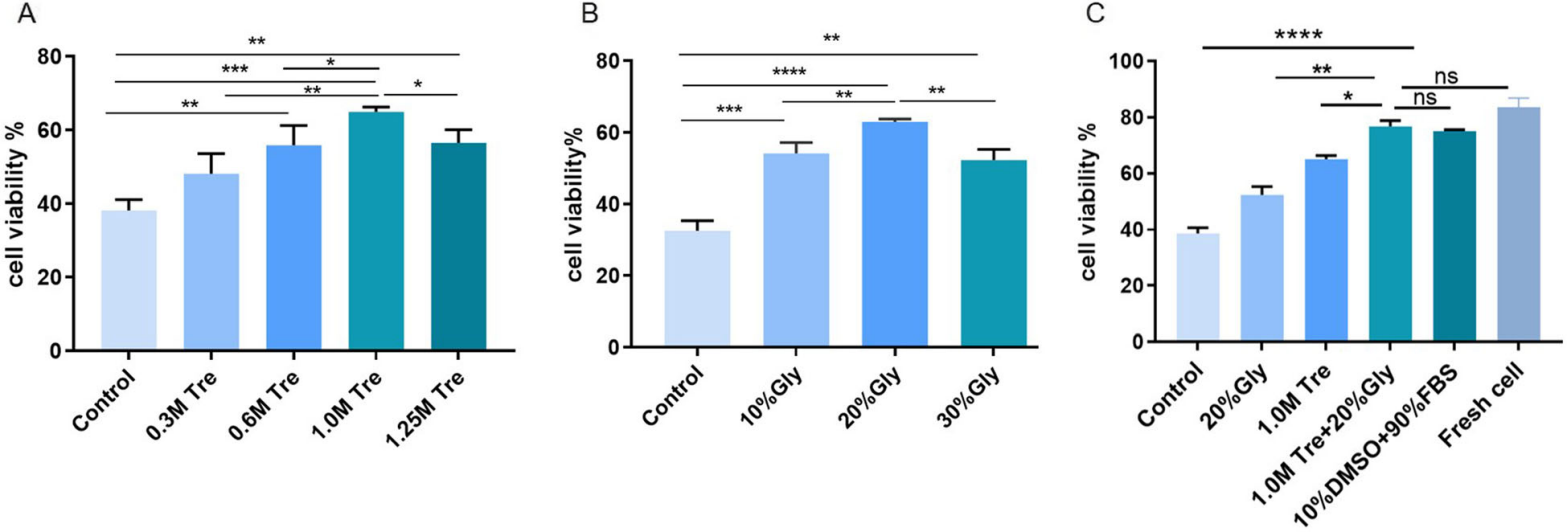
Effect of CPAs on the viability of post-thaw ADSCs. **a** Cell viability after cryopreservation in different concentrations of trehalose. **b** Cell viability after cryopreservation in different concentrations of glycerol. **c** Cell viability after cryopreservation in trehalose and glycerol. Values are expressed as the mean ± SD; *N* = 10. *p < 0.05; **p < 0.01; ***p < 0.001; ****p < 0.0001; ns, no significant difference

We assessed the effect of different concentrations of trehalose alone and glycerol alone on the viability of ADSCs. The viability of post-thaw ADSCs preserved in 1.0 M Tre alone (65 ± 1.10%) and 20% Gly (63 ± 0.65%) alone was significantly higher than that of those preserved in other concentrations (Fig. 1). We attempted to combine 1.0 M Tre and 20% Gly to cryopreserve the ADSCs. The viability results of the post-thaw ADSCs preserved in 1.0 M Tre + 20% Gly (77 ± 1.72%) did not differ from that of ADSCs preserved in 10% DMSO + 90% FBS (75 ± 0.37%, *p* = 0.2583) or fresh cells (82 ± 1.12%) and was significantly higher than the viability of the cells preserved in 1.0 M Tre alone (*p <* 0.05) and 20% Gly alone (*p <* 0.01).

### The CPA 1.0 M Tre + 20% Gly significantly improves the proliferation of post-thaw ADSCs compared to trehalose and glycerol alone, similar to 10% DMSO + 90% FBS

**Fig. 2:**
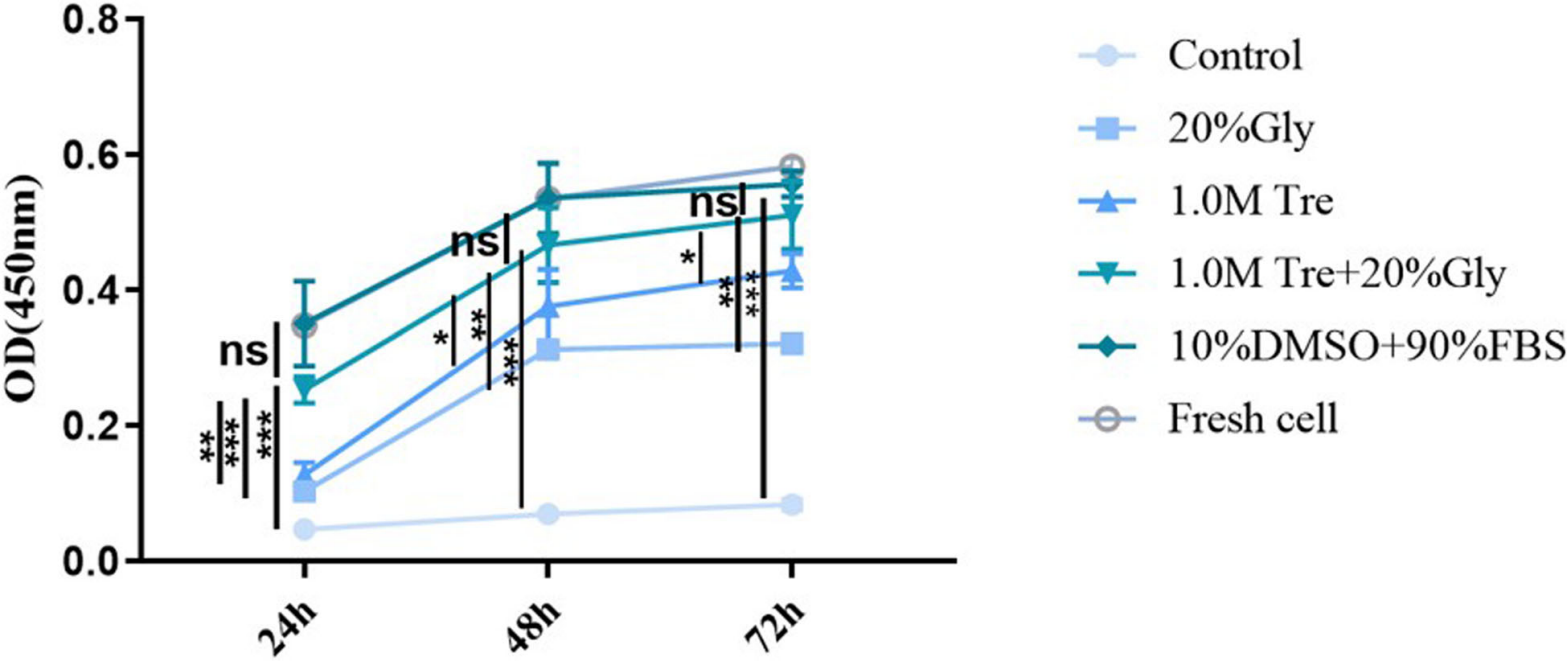
Effect of CPAs on the proliferation ability of post-thaw ADSCs. The proliferation of post-thaw ADSCs preserved in 1.0 M Tre + 20% Gly did not differ significantly from that of 10% DMSO + 90% FBS and fresh cells and was significantly higher than that of 1.0 M Tre and 20% Gly alone. Values are expressed as the mean ± SD; *N* = 10. *p < 0.05; **p < 0.01; **p < 0.001; ns, no significant difference

The proliferation of the post-thaw ADSCs preserved in 1.0 M Tre + 20% Gly (0.25 ± 0.02%, 24 h; 0.47 ± 0.05%, 48 h; 0.51 ± 0.04%, 72 h, Fig. 2) did not differ from those of the cells preserved in 10% DMSO + 90% FBS and fresh cells and was significantly higher than that of the cells preserved in 1.0 M Tre alone (*p <* 0.01) and 20% Gly alone (*p <* 0.001).

### ADSCs cryopreserved in 1.0 M Tre + 20% Gly exhibit a similar morphology compared to those in 10% DMSO + 90% FBS and fresh cells

**Fig. 3:**
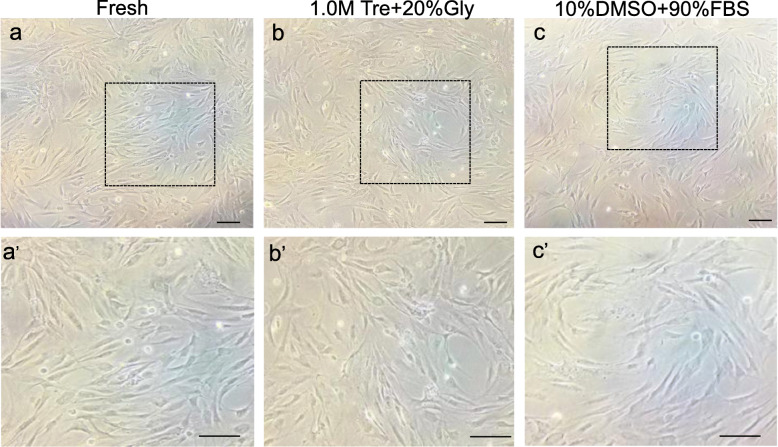
Effect of CPAs on the morphology of post-thaw ADSC. Cells cryopreserved in different CPAs were cultured for 72 h after thawing. **a** The morphology of fresh ADSCs. **b** The morphology of ADSCs preserved with 1.0 M Tre + 20% Gly. **c** The morphology of ADSCs preserved with 10% DMSO + 90% FBS. **a’**–**c’** The zoomed images of representative field of fresh ADSCs and ADSCs preserved with 1.0 M Tre + 20% Gly and 10% DMSO + 90% FBS. Scale bar = 100 μm

The morphology of the ADSCs preserved with 1.0 M Tre + 20% Gly or 10% DMSO + 90% FBS was documented after 72 h of culture and compared with that of fresh ADSCs. The results showed that there was no visible difference in the shape and cell density among these three groups (Fig. 3).

### The CPA of 1.0 M Tre + 20% Gly significantly improves the migration of post-thaw ADSCs compared to 10% DMSO + 90% FBS

**Fig. 4:**
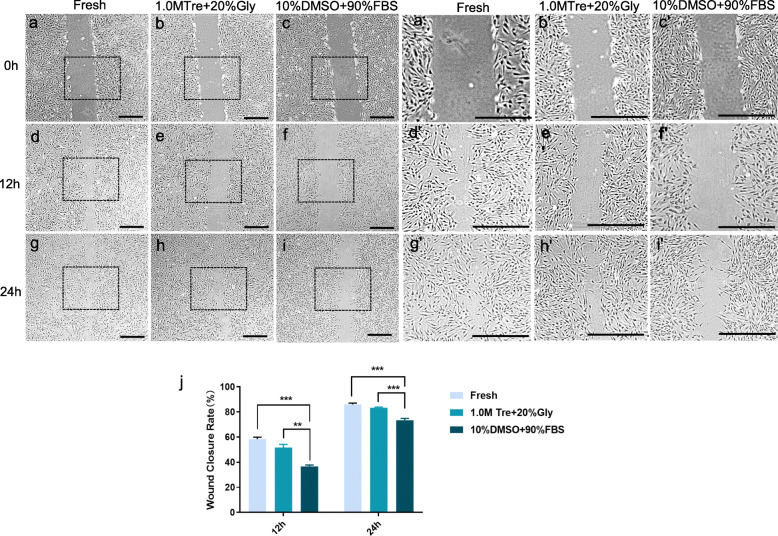
Effect of CPAs on the migration of post-thaw ADSCs. **a**–**c** Cells were scratched and photographed at time 0. **d**–**f** Photographs were taken again after 12 h and **g**–**i** 24 h. **a’**–**f’** Zoomed-in images of representative field of fresh ADSCs and ADSCs preserved with 1.0 M Tre + 20% Gly and 10% DMSO + 90% FBS at time 0 h, 12 h, and 24 h. **j** Quantification of the closure area is presented as the ratio of the closure area to the initial wound area. Values are expressed as the mean ± SD; *N* = 10. Scale bar = 100 μm. **p < 0.01; ***p < 0.001

The results of the migration of the post-thaw ADSCs preserved in 1.0 M Tre + 20% Gly (51 ± 2.05%, 12 h; 83 ± 0.47%, 24 h) did not differ from that of the fresh ADSCs and were significantly higher than that of the cells preserved in 10% DMSO + 90% FBS (37 ± 0.94%, *p <* 0.001, 12 h; 63 ± 1.25%, *p <* 0.001, 24 h) (Fig. 4).

### Cells cryopreserved in 1.0 M Tre + 20% Gly express the same surface markers as those in 10% DMSO + 90% FBS and fresh cells

**Fig. 5:**
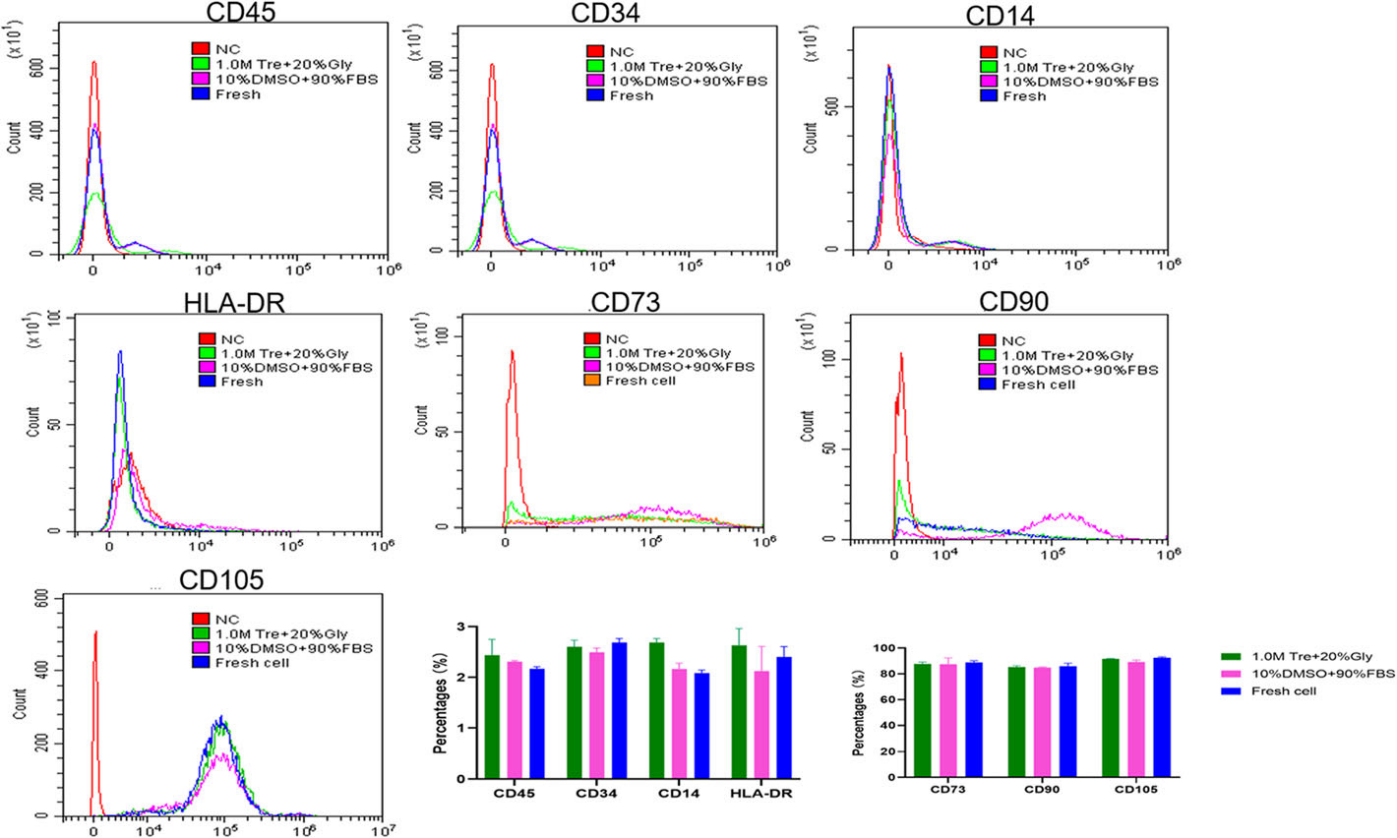
Expression of the surface markers after cryopreservation with different CPAs. The CPA of 1.0 M Tre + 20% Gly maintained the surface markers of ADSCs

ADSCs have positive markers CD73, CD90, and CD105 and negative markers CD45, CD34, CD14, and HLA-DR [16–18]. CD45, CD34, CD14, and HLA-DR expression was 2.44 ± 0.31%, 2.61 ± 0.13%, 2.11 ± 0.05%, and 2.63 ± 0.33%, respectively, in ADSCs from the 1.0 M Tre + 20% Gly group. The expression of CD73 in ADSCs in 1.0 M Tre + 20% Gly group was 84.38 ± 0.37% and that of CD90 was 85.32 ± 1.17% and that of CD105 was 91.71 ± 0.34%. The results showed that there were no differences in the expression of ADSC surface markers among the 1.0 M Tre + 20% Gly group, 10% DMSO + 90% FBS group and fresh ADSCs (all *p* > 0.05), demonstrating that the CPA of 1.0 M Tre + 20% Gly can maintain the surface markers of ADSCs (Fig. 5).

### Cells cryopreserved in 1.0 M Tre + 20% Gly exhibit the same multi-lineage differentiation potential as those in 10% DMSO + 90% FBS and fresh cells

**Fig. 6:**
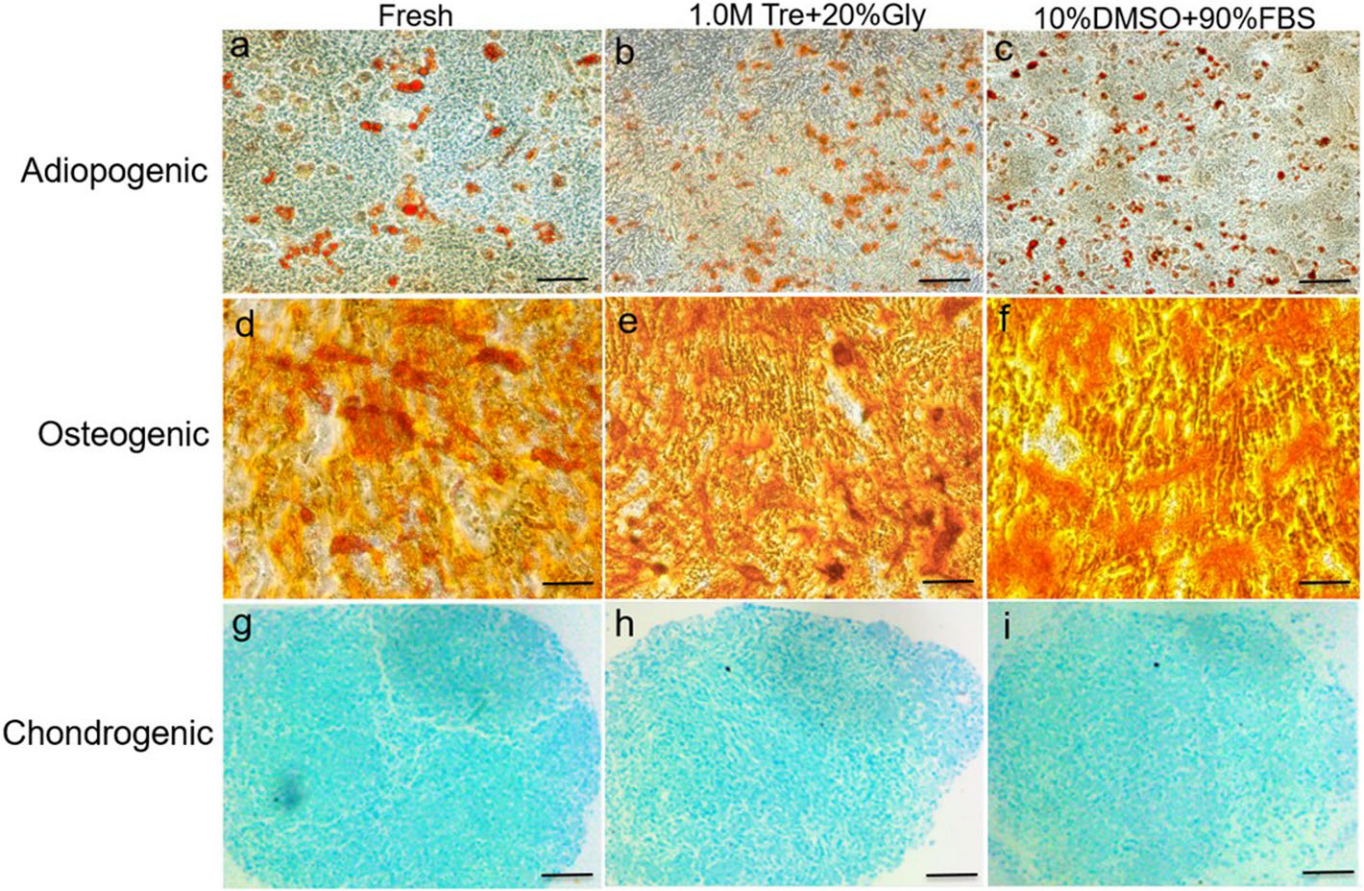
Comparable multi-lineage differentiation potential of ADSCs cryopreserved in different CPAs. **a**, **d**, **g** Adiopogenic, osteogenic, and chondrogenic differentiation potential of fresh cells. **b**, **e**, **h** Adiopogenic, osteogenic, and chondrogenic differentiation potential of cells cryopreserved in 1.0 M Tre + 20% Gly. **c**, **f**, **i** Adiopogenic, osteogenic, and chondrogenic differentiation potential of cells cryopreserved in 10% DMSO+ 90% FBS. Scale bar = 200 μm

To verify the multi-lineage differentiation potential of post-thaw ADSCs, adipogenic, osteogenic, and chondrogenic differential media were introduced to induce cell differentiation. The ADSCs cryopreserved in 1.0 M Tre + 20% Gly and 10% DMSO + 90% FBS presented the same adipogenic, osteogenic, and chondrogenic differentiation capacity as those preserved in 10% DMSO + 90% FBS and fresh ADSCs (Fig. 6). These results indicate that 1.0 M Tre + 20% Gly did not influence the adipogenic, osteogenic, or chondrogenic differentiation potential of the ADSCs.

## Discussion

Adipose-derived stem cells (ADSCs) are capable of self-renewal and multi-lineage differentiation and are considered an ideal type of stem cells for regenerative medicine [19, 20]. Various studies have shown that ADSCs have multiple functions in clinical applications, such as cartilage and bone repair, skin wound healing, neuronal regeneration, heart regeneration, and immune disorder treatment [21, 22]. The long-term preservation of ADSCs is crucial for clinical applications of cell-based therapy. Cryopreservation is commonly used to preserve cells, but cryodamage during the freezing process is a threat to cell viability [23]. CPAs help minimize the formation of ice crystals and other cryodamage by reducing the crystallization process of water and increasing the viscosity of the solution [13, 24, 25]. Currently, the most widely used CPA is DMSO combined with FBS. However, this CPA is not suitable for clinical applications due the risk of toxicity and zoonotic infection [24, 26–28]. Thus, the main purpose of current studies is to develop a non-toxic xeno-free CPA to fit the requirements of clinical applications.

Recently, many studies have investigated new CPAs for ADSCs; however, limitations exist. The current research mainly involves two approaches. The first approach is to reduce the concentration of DMSO. Shu et al. reported that using 0.5 M DMSO combined with 0.2 M trehalose achieve high efficiency in the cryopreservation of ADSCs [29]. López et al. reported that 3.5% DMSO + 3.5% ethylene glycol (EG) + 0.25 M trehalose + 2% poly (vinyl alcohol) (PVA) + 5% ficoll + 0.1 mM ethylene glycol-bis(2-aminoethyl ether)-N,N,N′,N′-tetraacetic acid (EGTA) achieved a better outcome in cell viability preservation than 10% DMSO + 90% FBS [30, 31]. However, these methods still contain DMSO, which is not suitable for clinical applications.

The other approach is to replace DMSO with other non-toxic reagents. Trehalose is a non-toxic and economic reagent for cryopreservation [32–35]. However, the mammalian plasma membrane is non-permeable to trehalose, limiting its efficiency [36]. When used separately, these reagents cannot reach the same outcome as 10% DMSO + 90% FBS [37]. Although several solutions have been reported to increase the permeability of trehalose, they are complex and difficult for clinical translation.

In this research, we evaluated the efficiency of a combination of trehalose and glycerol. When we used trehalose alone to preserve ADSCs, the viability of post-thaw ADSCs was 65 ± 1.10%, significantly lower than those of 10% DMSO + 90% FBS and fresh cell. It has been shown previously that glycerol can reduce the water crystallization process, inhibit the growth of ice crystal, and reduce damage to the cell structure and function [38]. However, the viability of cells cryopreserved in glycerol alone was also lower than that of cells cryopreserved in 10% DMSO + 90% FBS. In contrast, when trehalose was applied with glycerol, the viability of post-thaw ADSCs reached 77%, which is significantly higher than that achieved with either agent alone. Moreover, the proliferation rates of the cells preserved with 1.0 M Tre and 20% Gly were similar to those of the cells preserved with 10% DMSO + 90% FBS and fresh cells. The migration of the ADSCs treated with 1.0 M Tre and 20% Gly was significantly higher than that of the cells treated with 10% DMSO + 90% FBS, indicating that the combination of 1.0 M Tre and 20% Gly had a better effect in preserving cell functions.

Glycerol and trehalose, which are permeable and non-permeable CPAs, respectively, provide protection via different mechanisms. Trehalose can form a unique protective film on the cell surface, eliminate the formation of intra- and extracellular ice crystals, and prevent osmotic shock during freezing and thawing. Glycerol can weaken the crystallization process of water, increase the viscosity of the solution, and reduce the formation of intracellular ice crystals (IIF) [24, 26]. When combined, these two reagents could complement each other and provide better protection. Moreover, glycerol may help trehalose penetrate the cell membrane, improving the efficacy of trehalose.

Studies have shown that the duration of cryopreservation has minor influence on cell viability. Martinetti et al. showed that there was no difference in the viability of CD34+ cells cryopreserved for 20 days and for 3 months [28]. Moreover, Ntai et al. also reported that the time of preservation was no obvious influence on preservation. Using different CPAs to preserve pluripotent stem cells, the authors found that the viability did not differ between cells preserved for 7 days and those preserved for 30 days [39]. Therefore, we evaluated the outcome at 30 days of preservation in the present study.

Trehalose and glycerol, as a new CPA combination, are non-toxic and their safety has been proven in clinical applications. In the future, we will conduct numerous clinical trials to confirm our results.

## Conclusion

The combination of trehalose and glycerol can achieve a higher efficiency in ADSC cryopreservation than single reagent CPAs. The ADSCs preserved with 1.0 M Tre + 20% Gly showed high levels of cell viability, proliferation, migration, and multi-potential differentiation. As a non-toxic xeno-free CPA, 1.0 M Tre + 20% Gly may have promising clinical application prospects.

## Data Availability

The datasets used and/or analyzed during the current study are available from the corresponding author on reasonable request.

## Abbreviations

ADSCs: Adipose-derived stem cells
MSCs: Mesenchymal stem cells
CPA: Cryoprotective agent
DMSO: Dimethyl sulfoxide
FBS: Fetal bovine serum
Tre: Trehalose
Gly: Glycerol
(DMEM)/F12: Dulbecco's modified Eagle media: Nutrient Mixture F-12
EGTA: Ethylene glycol-bis(2-aminoethyl ether)-N,N,N′,N′-tetraacetic acid
PBS: Phosphate-buffered saline
CCK-8: Cell counting kit-8
EG: Ethylene glycol
PVA: Poly (vinyl alcohol)
